# Joint effect of uncertainty-of-outcome and calorie content on food preference

**DOI:** 10.1038/s41598-026-41632-x

**Published:** 2026-02-28

**Authors:** Lei Zheng, Yinqiang Yu, Pengfei Cheng, Shucheng Cao, Yun Yu, Zhen Yuan, Xueli Chen, Charles Spence

**Affiliations:** 1 School of Business, Macau University of Science and Technology, Taipa, Macao; 2 The Institute for Sustainable Development, Macau University of Science and Technology, Taipa, Macao; 3 Faculty of Medicine, Macau University of Science and Technology, Taipa, Macao; 4 Centre for Cognitive and brain Sciences and Ministry of Education Frontiers Science Center for Precision Oncology, University of Macau, Taipa, Macao; 5 School of Life Science and Technology, Xidian University, Xi’an, China; 6 Crossmodal Research Laboratory, Department of Experimental Psychology, University of Oxford, Oxford, UK

**Keywords:** Food preference, Uncertainty, Value comparison, Drift-diffusion model, EEG, Neuroscience, Psychology, Psychology

## Abstract

In today’s market, mystery boxes (where the contents are uncertain) introduce uncertainty concerning the outcome, offering the consumer the chance to receive one of several possible foods. However, the role of uncertainty-of-outcome induced in food preference is unclear. This research investigated how uncertainty-of-outcome affects food preference for high- versus low-calorie food products, and its cognitive and neural mechanisms. Fifty-eight participants completed a binary-choice task, deciding between a certain option (a known food) and an uncertain option (an unknown food hidden in a mystery box), and rated the expected tastiness (subjective value) of each food. The participants exhibited a stronger preference for uncertain options in the low- (vs. high-) calorie case. Using the drift-diffusion model, the drift rate was higher in the low- (vs. high-) calorie condition. Moreover, a larger frontal N2 amplitude and smaller frontal P3 and LPP amplitudes were detected in the low- (vs. high-) calorie condition during the binary-choice task. Besides, frontal LPP amplitudes were negatively correlated with drift rate, suggesting that more cognitive effort was required to accumulate evidence to make a decision. Additionally, high-calorie foods elicited larger frontal alpha event-related desynchronization than low-calorie foods, thus suggesting participants require more evidence and effort in value comparison during the decision-making. This research highlights how uncertainty-of-outcome enhances the reward value of food, especially low-calorie foods, and thus impacts food preference, providing insights for developing marketing and public health strategies.

**Supplementary Information:**

The online version contains supplementary material available at 10.1038/s41598-026-41632-x.

## Introduction

Although the World Health Organization (WHO) recommends a healthy diet, healthy foods are often considered to be tasteless and boring^1,2^. Based on the notion of “eating first with our eyes”^3,4^, the uncertainty marketing strategy, whereby marketers deliberately withhold information in order to capture the attention and interest of consumers is believed to increase the food’s appeal and positive expectations^5,6^. By strategically incorporating uncertainty-of-outcome into product promotions, marketers can help to create a sense of mystery for people^6^, thus enhancing purchase intention^7^. Hence, the first research goal was to investigate the effect of uncertainty-of-outcome on food preference and explore its underlying neural mechanism. Moreover, since high-calorie foods are generally more appealing than low-calorie foods, uncertainty-of-outcome may play a more crucial role in enhancing the perceived tastiness of low-calorie foods, which are often considered tasteless. Thus, the second research goal was to study whether the effect of uncertainty-of-outcome on food preference would differ between high- and low-calorie foods.

### Food preference and uncertain promotion

Uncertain promotions, conveying uncertainty-of-outcome to consumers, are now widespread, even permeating the food industry. Brands such as Oreo, Pringles^8^, and Fanta^9^ exemplify this trend with their increasing releases of mystery-flavored products. These brands incorporate uncertainty-of-outcome directly into their product promotions, creating food mystery boxes/cans that contain an unknown, randomized product. Although conveying certainty through packaging, such as using transparent materials or clearly labeling specific flavors, can enhance consumer trust in the quality of the product^10^, uncertainty-of-outcome triggers mystery and curiosity^6^, leading to optimistic expectations^11,12^. Notably, positive expectations have been linked to overestimating the probability of favorable outcomes^13^ and optimistic interpretations of receiving a good outcome in uncertain promotions^12^. Accordingly, uncertainty-of-outcome is thought to motivate people to seek pleasure and engage in hedonic consumption^14^. Surprisingly, even when anticipating negative outcomes, people tend to prefer the uncertain option due to their inherent curiosity and urge to resolve uncertainty^6^. Thus, using uncertainty-of-outcome in food promotions (e.g., labeling random flavors and ingredients on food packaging) may increase expectations of tastiness, perhaps by heightening physiological arousal and enhancing eye appeal^11,15,16^.

The dual-process theory posits that human behaviour is governed by two distinct systems: one that is automatic, fast, and unconscious, and another that is reflective, slow, and cognitively effortful^17,18^. In the context of food choices, high-calorie foods are often perceived as more palatable and rewarding, which can activate automatic approach tendencies driven by the automatic system^19,20^. For low-calorie foods, given that people tend to have lower taste expectations for low-calorie foods, using strategies such as using specific containers or packaging is considered an effective way to enhance their perceived tastiness^1,21^. Uncertainty-of-outcome, by altering wanting processes and driving motivational excitement, can lead people to develop optimistic expectations regarding the tastiness of a food product^11,12,14^. Specifically, previous studies have demonstrated that uncertainty-of-outcome increases perceived fun^16^ and positive surprise^15^, resulting in increased consumption^7^. Thus, uncertainty-of-outcome may modulate these processes by enhancing motivational salience^11^ and generating optimistic expectations regarding food tastiness, thereby increasing the subjective reward value of the options. Since the consumer’s preference for high-calorie foods is rooted in biology^20,22^, the effect of uncertainty-of-outcome on food preference may be stronger for low- (vs. high-) calorie foods, given that low-calorie foods are generally considered to be less appealing.

### Value-based decision-making

According to the appetitive brain network^2,23,24^, the amygdala and the orbitofrontal cortex (OFC) process visual food sensory input, encoding its subjective reward value and relaying this information to decision-making regions, where it is further modulated by the dorsolateral prefrontal cortex (PFC) through cognitive control. In other words, food preference represents a value-based decision-making process whereby people perceive, recode, and compare reward values in order to guide their food choices. Particularly, high-calorie foods are often more pleasant to consume than low-calorie foods such as vegetables^25,26^. This is because the perceived hedonic or reward value of food is strongly impacted by its calorie content^26^, which is a crucial driver of consumer visual attention and eating behaviour^4,27^. Uncertainty-of-outcome is considered to play a significant role in activating the reward-processing system in the human brain^28–30^. In particular, functional MRI has demonstrated the role of the OFC and the amygdala in evaluating uncertain reward^28,31^, and the anterior cingulated cortex (ACC) has been implicated in outcome evaluation^32^.

People often compare food items when making decisions about what to eat, with reward processing playing a pivotal role. Specifically, people often engage in value-based decision-making by accumulating and evaluating evidence related to potential rewards to reach a decision^33^. Accordingly, several brain regions, including the PFC, OFC, and amygdala, are thought to be involved in processing the subjective reward value of food^2,23,24^. Electroencephalography (EEG), using techniques such as event-related potentials (ERPs) and event-related oscillations (EROs), helps to unveil rapid brain dynamics that cannot currently be captured by fMRI and PET. In fact, value-related EEG signals appear within 200 ms of stimulus onset in the binary-choice task^34,35^. According to Rangel^36^, people tend to assign values to different options and engage in a comparative evaluation of those values before making a selection. The process of value-based preference formation is reflected in a progressive pattern of brain activation, originating in occipito-temporal cortical areas to fronto-central sites over the course of the ERPs^34,37^.

### ERPs and EROs in food preference

Value-based food choice is a dynamic process involving multiple stages, including conflict detection, evaluation, and reward anticipation. Each of these stages is considered to be linked to distinct ERPs and EROs^37–40^. In the binary-choice task, conflict arises especially when the two options are very similar and thus difficult to distinguish^41,42^. Specifically, hard-to-discriminate stimuli often elicit larger N2 amplitudes^43^. Particularly, the frontal N2 plays a significant role in the processing of economic value^44^ and during food-related cognitive control^37^, with larger N2 amplitudes observed for those items having a low subjective value^34^. Consistent with this finding, larger N2 amplitudes were reported for two loss options compared to two gain options^45^.

In addition to early conflict detection, later ERPs are primarily associated with value evaluation and outcome encoding^34,36,37,46^. Both the P3 and the Late Positive Potential (LPP) are sensitive to reward valence and magnitude, and their amplitudes scale with the subjective value of the options under consideration^39,42,47^. Specifically, the P3 reflects outcome evaluation during decision-making, with its amplitude varying based on reward probability, significance, and magnitude^34,47^. In the delay-discounting task, P3 amplitudes reflect the cost-benefit evaluation process in terms of the value of both options^48^. Similarly, larger fronto-central LPP amplitudes were observed for high-calorie foods (e.g., meat and sweets) compared to low-calorie foods (e.g., vegetables), suggesting a relationship with food reward magnitude^40^. Harris, et al.^49^ observed frontal late ERP activity (450–650ms post-stimulus) in response to value-based decision-making processes, and the LPP is thought to represent the accumulation of evidence during decision-making^50^. Hence, both P3 and LPP are thought to reflect cognitive processes in terms of both reward valence and value^49^.

According to time-locked ERPs, reward anticipation is also represented in low-frequency EROs^51^. Among them, alpha power is regarded as an inverse index of cortical activity^52^, reflecting the control of cortical excitability in the accumulation of sensory evidence^53^. As the frontal brain region is recognized for its critical role in encoding and processing subjective value^28,31,32^, frontal alpha activity is thought to serve an inhibitory role in subjective value coding^54^. Actually, the suppression of frontal alpha activity (Event-Related Desynchronization, ERD) is thought to reflect active bottom-up information processing, whereas frontal alpha’s synchronization (Event-Related Synchronization, ERS) reflects top-down processing^55^. Interestingly, alpha activities are modulated by subjective value, exhibiting a negative relationship where power decreases as perceived value increases^51,56^. Particularly, alpha ERD is related to increased approach motivation and reward processing^40,42^. According to HajiHosseini and Hutcherson^53^, frontal alpha ERD reflects sensory evidence accumulation and food tastiness evaluation.

### The present study

Although the role of food calorie content and the influence of uncertainty and curiosity in decision-making^6,37,39^ has been examined in previous research, the role of uncertainty-of-outcome induced in food preference, as well as the underlying cognitive and neural mechanisms, remains unclear. Following up on previous binary-choice studies^34,44^, the present research adopted the Hierarchical Drift-Diffusion Model (HDDM) to explore the cognitive mechanism underlying food decision-making^57^. The model includes four cognitive parameters: drift rate (accumulating decision evidence), starting point (starting bias before decision), decision boundary (risky or conservative decision strategy), and non-decision time (perceptual and motor processes). We focused on the drift rate and starting point to understand the value-based decision-making process. Prior research suggests external factors have a greater impact on the subjective value of low-calorie foods as compared to high-calorie foods, possibly because the inherent reward value of high-calorie foods is already high and thus less susceptible to external manipulation^1,2^. Given the subjective value caused by the uncertainty-of-outcome and high-calorie foods^1,6,12^, participants may prefer uncertain (vs. certain) options in low-calorie condition (i.e., larger drift rate and starting point), compared to in high-calorie condition. Additionally, frontal ERPs, specifically the N2, P3, and LPP, reflect subjective value processing during food decision-making^35,37,46^. Besides, frontal alpha ERD is considered to reflect the evidence accumulation and subjective value processing in dietary decision making^51,53,56^. Thus, these frontal ERPs and alpha ERD were analyzed in order to reveal the neural activity during the food preference.

## Methods

### Participants

The sample size for this study was determined based on prior research on binary choices (Cohen’s *d* = 0.37), specifically focusing on preferences between certain and uncertain options^6^. Using a within-subjects design (paired-samples t-test), α = 0.05 and power = 0.80, a minimum sample size of 60 was estimated using G*Power (version 3.1.9.7)^58^.

Sixty-five participants from two Chinese universities were recruited through flyers posted on WeChat groups and moments (WeChat is a popular social media platform in China). The eligibility criteria for participation were as follows: (1) ≥ 18 years of age, (2) right-handed, (3) normal/corrected-to-normal vision, (4) no psychiatric/neurological disorder history, (5) no weight loss goals, (6) not vegetarian, and (7) women not on their menstrual period. Seven participants were excluded due to excessive artifact movements, resulting in a final sample of 58 participants (31 Women; Age = 22.12 ± 2.87 years; BMI = 22.10 ± 3.22 kg/m^2^). The research was conducted in accordance with the Declaration of Helsinki. All participants provided written consent. The research protocol was approved by the Ethics Committee of Macau University of Science and Technology and pre-registered on the Open Science Framework (OSF; https://osf.io/bhx2j/overview). After the research was completed, participants were debriefed and thanked with ¥100 RMB (≈ $15 USD).

### Procedure and materials

The experiment adopted an one factor (high- vs. low-calorie) within-subject design. Participants were instructed to refrain from eating or drinking anything except water for at least three hours prior to their appointment. The experiment consisted of three tasks: a food rating task, a food choice task, and a healthiness rating task, all tasks developed using E-Prime 3.0. The food images, selected from a standardized food-picture database^59^, included 36 high- and 36 low-calorie foods. Twelve images (6 high- and 6 low-calorie foods) were used for training trials, and 60 for the main experiment. High- and low-calorie food images showed non-significant differences in image brightness (*t*[58] = − 0.56, *p* = 0.580), visual contrast (*t*[58] = 0.43, *p* = 0.667), and subjective complexity (*t*[58] = − 0.32 *p* = 0.749). Participants then provided their demographic information.

### Task

**Fig. 1:**
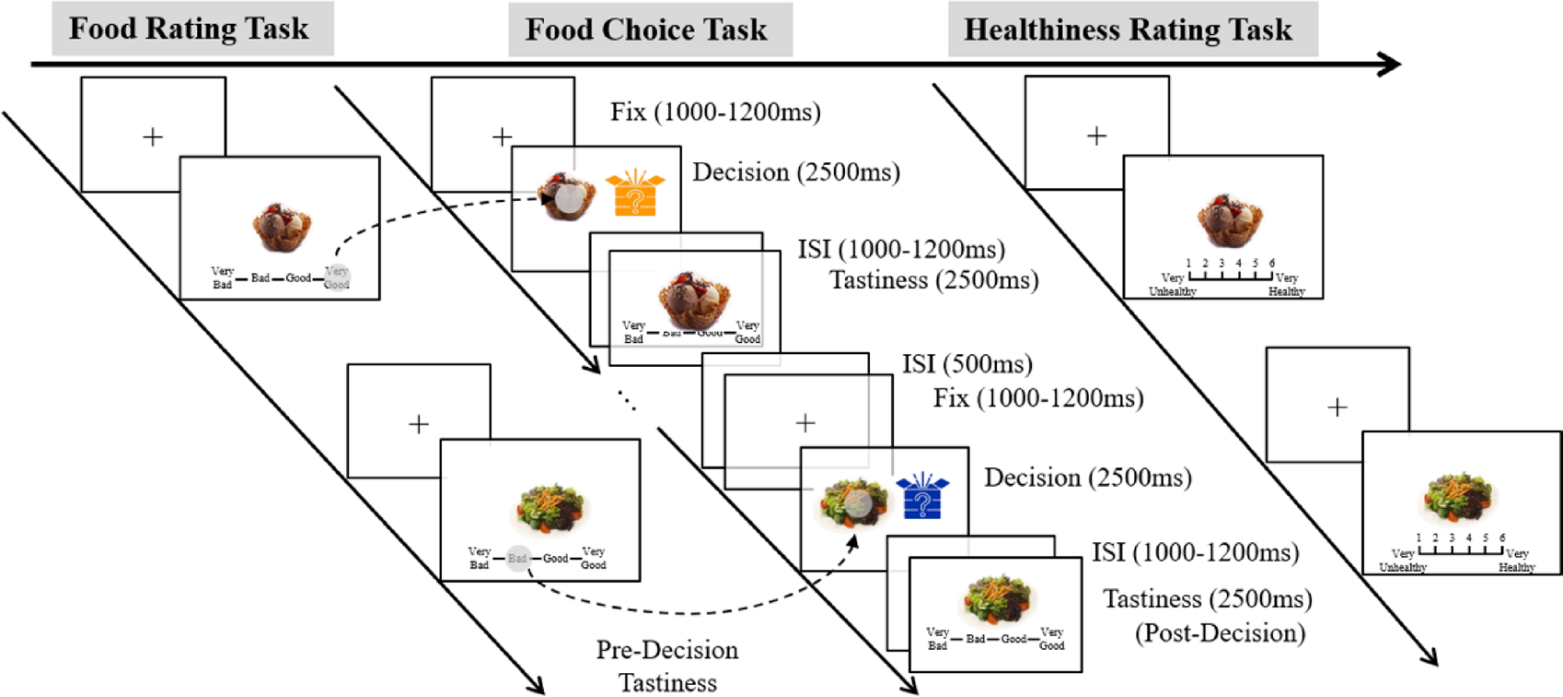
Research procedure. This research includes three tasks: food rate task, food choice task, and healthiness rating task. Participants first rated the tastiness of food images on a 4-point scale (1 = Very Bad, 2 = Bad, 3 = Good, 4 = Very Good). Next, the participants were instructed to complete the food choice task. Each trial began with a fixation cross (1000–1200 ms), followed by the simultaneous presentation of the certain and uncertain food options. Participants had 2500 ms to choose which they would eat (EEG recorded). After an ISI (1000–1200 ms), the chosen option was displayed. If the uncertain option was selected, a high- or low-calorie food image was revealed. If the certain option was selected, the chosen food image was re-displayed. Participants then rated tastiness (1–4 scale; Very bad to Very good) within 2500 ms. Finally, in the healthiness rating task, the participants rated the healthiness of each food on a 6-point scale (1 = Very unhealthy to 6 = Very healthy).

Participants first completed the food-rating task, in which they rated the tastiness of 60 randomly presented food images on a 1–4 scale (from very bad to very good). Next, participants completed the food choice task, selecting the food they were “willing to eat” (F for left, J for right) from two choices: one was a certain option (known food presented on the screen), and the other was an uncertain option (unknown food hidden inside a mystery box). The task included a training block (6 trials) and two 30-trial experimental blocks (high- and low-calorie). The order of the calorie blocks was counterbalanced across participants to control for potential order effects. During the training block, participants were instructed to choose between certain and uncertain options on each trial. Each block consisted of either high-calorie or low-calorie food items. All the food options were selected from the database used in the food rating task. The presentation of certain and uncertain options (left vs. right) was counterbalanced in each block. As shown in Fig. 1, in each trial, participants were instructed to focus on a fixation cross at the center of the screen (1000–1200 ms). Next, two items (a certain and an uncertain option) appeared on the right and left sides of the screen. The participants were instructed to make decisions within 2500 ms, deciding which of the two displayed items they were willing to eat, while their EEG was recorded. Following a 1000–1200 ms interstimulus interval (ISI), the selected food appeared at the center of the screen. If the uncertain option was selected, it was replaced by a high- or low-calorie food. Each one of the food images was displayed once. The participants then rated the tastiness of their selected item on a 1–4 scale (very bad to very good; pressing D, F, J, or K), within 2500 ms. Finally, the participants rated the healthiness of each food on a 1-6 scale (very unhealthy to very healthy; *M*_*low*_ *=* 5.42 ± 0.69 vs. *M*_*high*_ *=* 2.87 ± 0.88; *t*[57] = 16.45, *p* < 0.001, Cohen’s *d* = 2.16).

### EEG data acquisition

EEG data were recorded using the Neuroscan system with 64 Ag/AgCl scalp electrodes according to the international 10–10 System. The ground electrode was located between FPz and Fz, and the reference electrode was located at Cz. The EEG data were amplified by a Neuroscan SynAmps 2 amplifier with a sampling frequency of 500 Hz. The impedance for each electrode was kept below 5 kΩ. An online bandpass filter from 0.05 Hz to 100 Hz were applied to avoid the interference with baseline drift and high frequency noise. The participants were required to keep quiet during EEG acquisition. Moreover, four additional electrodes were attached below and above the left eye as well as at the bilateral outer canthi to record horizontal and vertical electrooculogram (EOG).

### EEG offline analysis

EEG recordings were preprocessed offline using MATLAB. The raw data was re-referenced offline to the bilateral mastoids (M1, M2), and filtered with a bandpass filter from 0.1 to 30 Hz. EEG deflections resulting from horizontal eye movements and eye blinks were corrected using the independent component analysis with the EOG data. EEG data were segmented in epochs: from 200 ms before to 800 ms after options onset for ERP analysis, and from 1000 ms before to 2000 ms after options onset for time-frequency analysis. Epochs with amplitudes exceeding ± 100 µV and/or visual artifacts were rejected. Lastly, epochs were baseline-corrected with the activity from − 200 to 0 ms relative to options onset. After the preprocessing, a total of 28.37 ± 1.24 trials of every participant in each condition remained for subsequent EEG data analysis. Since frontal electrodes are considered to be sensitive to food-reward evaluation and value-based decision-making^2,37,40,53^, fronto-central electrode cluster (F1, Fz, F2, FC1, FCz, FC2) was extracted for EEG analyses.

The ERP components, including N2 (200–300 ms), P3 (300–400 ms), and LPP (500–700 ms), were selected based on previous food-related studies^39,60^ and visual inspection of the grand-averaged ERPs. Mean amplitudes were calculated by averaging the amplitude values within their respective time windows for each condition. Both clustered ERP mean values and individual electrode ERPs were reported. Although P3 and LPP components are often maximal at parietal sites in other reward-related tasks^37,40^, the current study focused on fronto-central activity due to its relevance to subjective value processing.

The time-frequency analysis was conducted using the Morlet wavelet convolution with a sliding Hanning tapered window. The signal length of this sliding window is 500 ms, and the step length is 10 ms. The time-frequency power was normalized by dividing the average power from − 800 ms to − 400 ms. In each condition, overall power was computed by averaging single-trial power within each condition. To investigate neural oscillations related to the food-related decision-making, a cluster-based permutation *t*-test was conducted within the 5–30 Hz during the 0–1,000 ms time window.

### Hierarchical drift-diffusion model (HDDM)

To investigate the cognitive mechanisms underlying decision-making, HDDM was used to analyze choice and response time^57^. The model uses a hierarchical Bayesian approach to estimate each participant’s parameters from a group distribution, with joint posterior distributions estimated via the Python HDDM toolbox^61^. The Markov Chain Monte Carlo (MCMC) method was used to estimate the hierarchical Bayesian parameters. After excluding trials with the longest response times (5%), four MCMC chains were run, each generating 11,000 samples. To ensure convergence, the initial 1,000 burn-in samples were discarded, resulting in a joint posterior distribution estimate for all parameters using the remaining 40,000 samples. Convergence was assessed using the Gelman-Rubin R statistic, with values near 1 indicating reliable model fitting^62^. According to the model comparison, both the deviance information criterion (DIC) and posterior prediction check (PPC) were used, with lower DIC values and mean square errors of PPC indicating better model fit. Best-fitting parameters were averaged across all chains’ posterior distributions.

In this research, the “choose uncertain option” and “choose certain option” options in each trial were set as the upper and lower decision boundaries, respectively. In the HDDM, two parameters—drift rate and starting point—were allowed to vary across conditions. We also tested single-parameter model and four-parameter model, as well as conducted model comparisons to select the best-fitting model using DIC and PPC. Besides, HDDM regressions were conducted to examine the effect of food tastiness and neural activity on the hierarchical Bayesian parameters.

## Results

### Effects of uncertainty-of-outcome and calorie content on food preferences

**Fig. 2:**
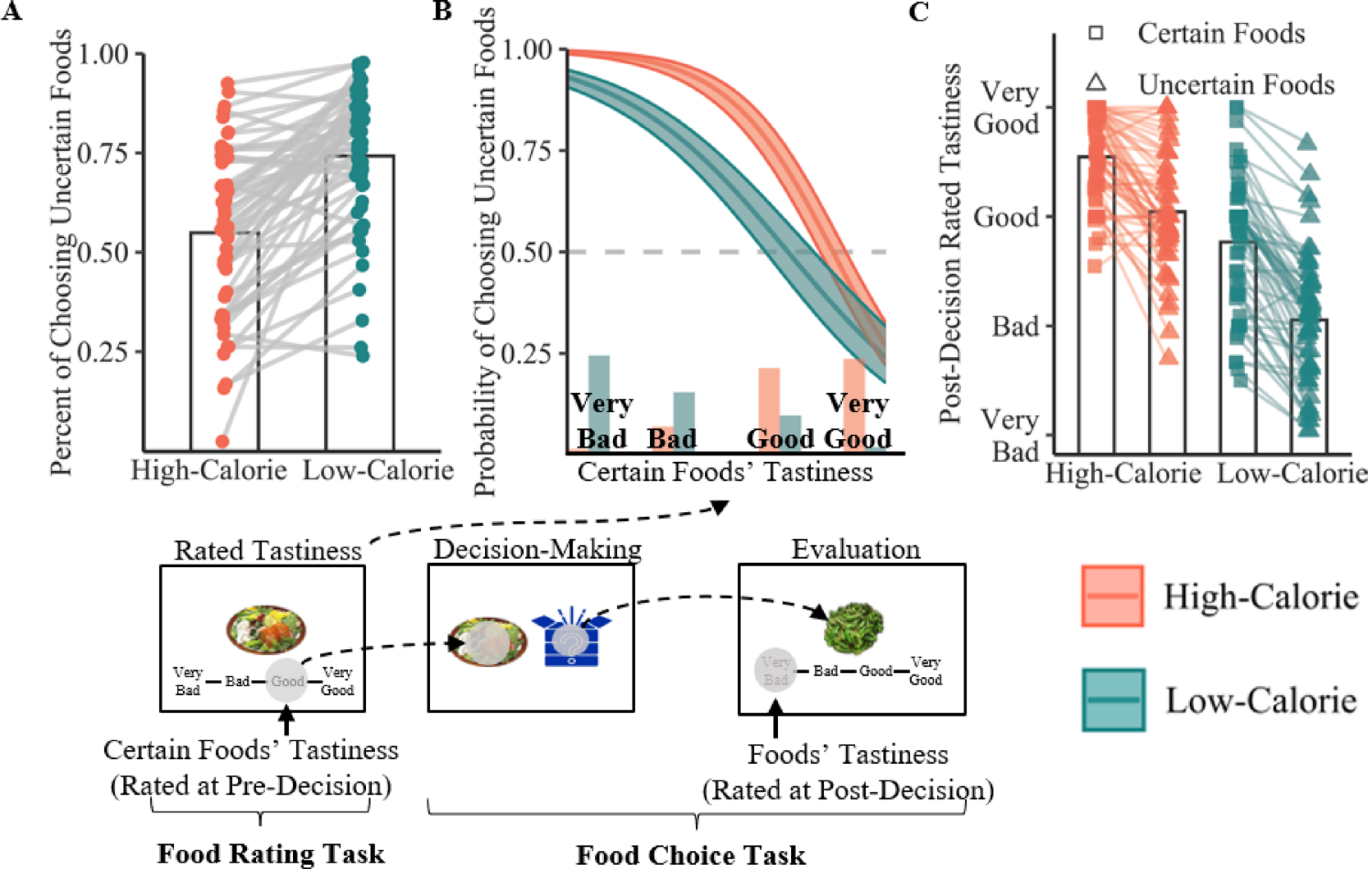
Joint effect of calorie content and uncertainty-of-outcome on food preference. (**A**) Participants exhibited a greater preference for the uncertain option more for low-calorie foods than for high-calorie foods. (**B**) The probability of selecting the uncertain option decreased with increasing tastiness of the certain option, particularly for high-calorie foods. (**C**) When participants were instructed to report their tastiness towards the food after decision-making, they rated uncertain options as less tasty than certain options, regardless of calorie condition.

To examine how uncertainty-of-outcome and calorie content influenced choice behaviour, the selection rate of the uncertain option in the food choice task was analyzed. Participants showed a stronger preference for the uncertain option in the low-calorie condition (M = 74.30 ± 17.53%) compared to the high-calorie condition (M = 54.89 ± 20.67%), indicating an interaction between uncertainty-of-outcome and calorie content (*t*[57] = 8.18, *p* < 0.001, Cohen’s *d* = 1.07; Fig. 2A). The selection rate of the uncertain option remained stable throughout the rounds, fluctuating around the mean value (Supplementary Fig. 1), suggesting consistent food preferences over time.

Multilevel logistic regression models were conducted to assess how the perceived tastiness of certain option (uncentered) and calorie content (1 = low-calorie, 0 = high-calorie) impacted preferences for food choice (1 = choose uncertain option, 0 = choose certain option). The results revealed a significant interaction between perceived tastiness of certain option and calorie content on preferences for food choice (*B* = 0.76, *SE* = 0.14, 95% CI [0.49, 1.02], *p* < 0.001; Fig. 2B). According to the simple effect analysis, when the certain option was perceived as higher in tastiness, participants were less likely to choose the uncertain option. This effect was stronger in the high-calorie condition (Intercept = 8.40; B = − 2.41, SE = 0.14, 95% CI [− 2.68, − 2.14], *p* < 0.001) than in the low-calorie condition (Intercept = 5.38; B = − 1.93, SE = 0.13, 95% CI [− 2.19, − 1.67], *p* < 0.001). Therefore, although Fig. 2A showed a general preference for the uncertain option, particularly in the low-calorie condition, regression analyses indicated that when accounting for the moderating role of the perceived tastiness of the certain option, participants were more likely to choose the uncertain option in the high-calorie than in the low-calorie condition.

Surprisingly, in the post-decision stage, uncertain options were rated as less tasty than certain options for both calorie conditions (*F*[1, 57] = 115.65, *p* < 0.001, ${\eta}_{p}^{2}$ = 0.67). Specifically, as shown in Fig. 2C, participants consistently rated uncertain options as less tasty for both high-calorie (M_*certain*_ = 3.60 ± 0.56 vs. M_*uncertain*_ = 2.95 ± 0.94; *F*[1, 57] = 144, *p* < 0.001, ${\eta}_{p}^{2}$ = 0.72) and low-calorie conditions (M_*certain*_ = 2.83 ± 0.79 vs. M_*uncertain*_ = 1.99 ± 0.88; *F*[1, 57] = 66, *p* < 0.001, ${\eta}_{p}^{2}$ = 0.54). Additionally, the effect of the certain option’s tastiness on the likelihood of choosing the uncertain (vs. certain) option was moderated by the uncertain option’s post-decision tastiness in the previous round (*B* = − 0.18, *SE* = 0.06, 95% CI [− 0.30, − 0.05], *p* = 0.006; Supplementary Fig. 2). Specifically, when the uncertain option (previous round) was rated higher in terms of perceived tastiness, the negative influence of the certain option’s perceived tastiness on choosing the uncertain option in the current round became weaker.

Analysis of response times (RTs) revealed an interaction between uncertainty-of-outcome and calorie content (*F*[1, 57] = 12.11, *p* < 0.001, ${\eta}_{p}^{2}$ = 0.18; Supplementary Fig. 3). Specifically, the participants were faster to choose uncertain options in the low-calorie condition (*F*[1, 57] = 17.8, *p* < 0.001, ${\eta}_{p}^{2}$ = 0.24; M_*certain*_ = 1.14 ± 0.41 s vs. M_*uncertain*_ = 1.07 ± 0.36 s), with no significant difference reported in the high-calorie condition (*F*[1, 57] = 1.22, *p* = 0.274, ${\eta}_{p}^{2}$ = 0.02; M_*certain*_ = 1.14 ± 0.39 s vs. M_*uncertain*_ = 1.13 ± 0.36 s). Furthermore, a multilevel regression revealed no initial response time difference between high- and low-calorie foods, but decision-making was relatively faster for low- (vs. high-) calorie foods in later rounds (Trial ✕ Calorie content: *B* = − 0.02, *SE* = 0.01, *t* = 1.98, *p* = 0.048; Supplementary Fig. 4).

### Effects of uncertainty-of-outcome and calorie content on computational model parameters

**Fig. 3:**
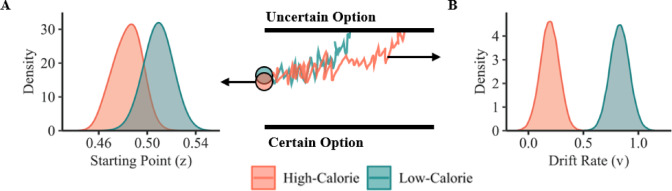
The effects of calorie content on computational model parameters in response to uncertain versus certain options. (**A**) The starting point (*z*) did not differ significantly between high- and low-calorie foods, indicating no initial bias towards either calorie condition. (**B**) Drift rate (*v*) was significantly higher in the low- (vs. high-) calorie condition, suggesting faster decision-making favoring uncertainty in this condition.

HDDM was used to investigate the cognitive mechanisms underlying participants’ food choices (1 = choose uncertain option, 0 = choose certain option) under conditions of uncertainty-of-outcome and varying calorie content (1 = low-calorie, 0 = high-calorie). The results showed that there was a non-significant difference in starting bias between high- and low-calorie foods (*z*_*diff*_ = 0.02, 95%HDI [− 0.004, 0.05], Fig. 3A). However, the posterior probability distribution of drift rate was larger when choosing uncertain options in the low- (vs. high-) calorie condition (Fig. 3B; *v*_*diff*_ = 0.64, 95%HDI [0.50, 0.77]). HDDMs were also conducted with either four parameters (i.e., *v*,* z*,* a*, and *τ*) or the single drift-rate parameter. The results of drift rate and starting point were consistent across models (see Supplementary Fig. 5 and Supplementary Table 1), suggesting that participants attributed higher value to uncertain (vs. certain) options in the low-calorie condition, rather than showing biased responses, compared to the high-calorie condition.

Additionally, HDDM regression model revealed that certain option’s tastiness significantly interacted with calorie content in predicting drift rate (*B* = − 0.90, 95%HDI [− 1.01, − 0.78]), but not starting point (*B* = 0.01, 95%HDI [− 0.03, 0.02]). As the perceived tastiness of certain options increased, the participants were more likely to choose the certain option, making decisions to select the uncertain option more difficult and slowing the rate of evidence accumulation in the high-calorie condition (*B* = − 1.62, 95%HDI [− 1.79, − 1.46]), compared to the low-calorie condition (*B* = − 1.31, 95%HDI [− 1.48, − 1.14]).

### Effects of uncertainty-of-outcome and calorie content on neural activity

**Fig. 4:**
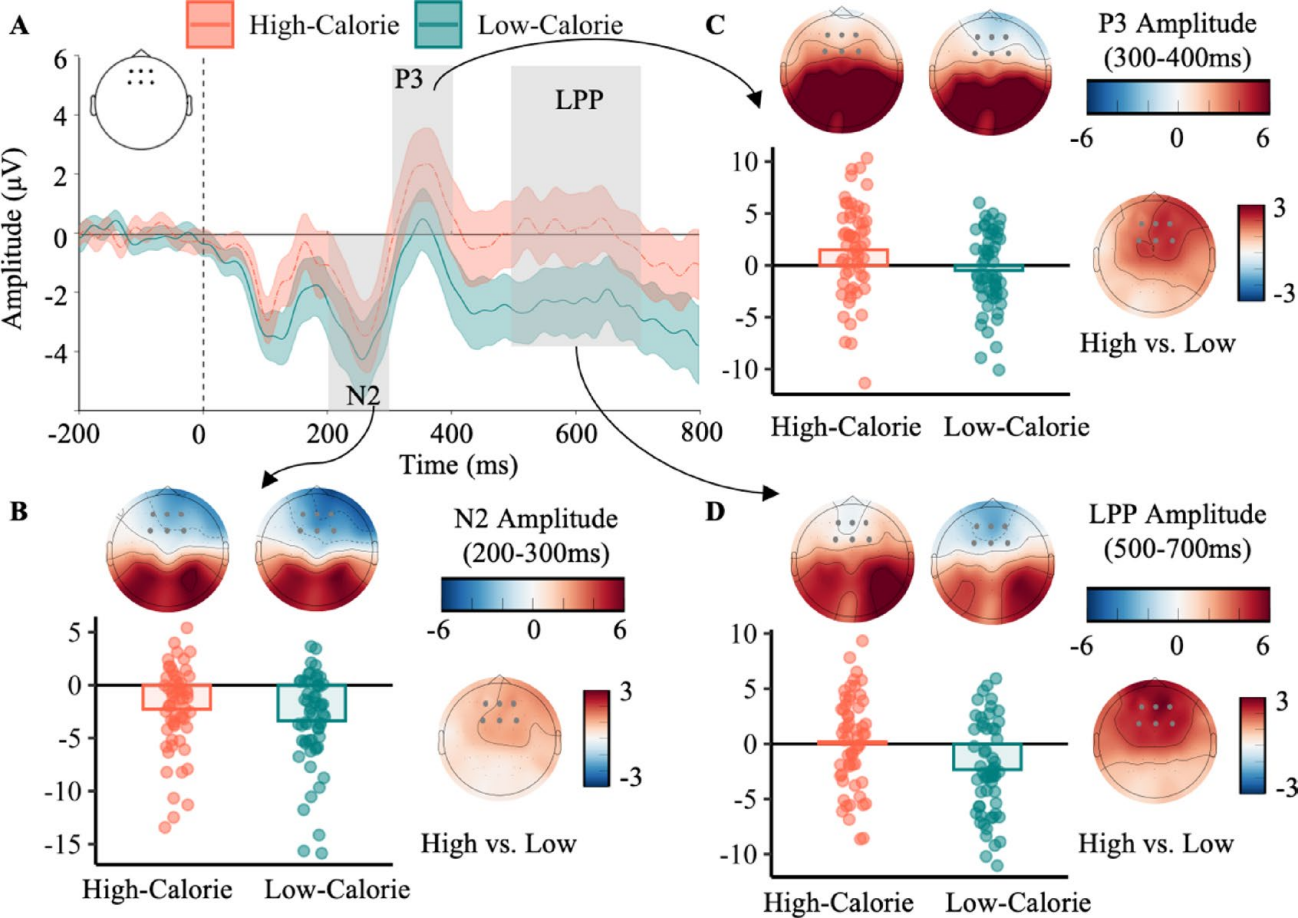
Amplitude differences of frontal event-related potentials between high- and low-calorie conditions. (**A**) Event-related potentials (ERPs) at fronto-central electrodes for high-calorie (red) and low-calorie (green) conditions. (**B**) Topographic maps of the N2 component are shown for both high- and low-calorie conditions, along with the difference wave (high-calorie minus low-calorie). (**C**) Topographic maps of the P3 component are presented for both high- and low-calorie conditions, along with the corresponding difference wave (high-calorie minus low-calorie). (**D**) Topographic maps of the late positive potential (LPP) are presented for both high- and low-calorie conditions, along with the difference wave (high-calorie minus low-calorie).

As shown in Fig. 4A, the effects of calorie content on neural activity during food-related decision-making were examined, specifically focusing on ERP components (N2, P3, and LPP) at the fronto-central regions (F1, Fz, F2, FC1, FCz, FC2). Low-calorie foods (M = − 3.37 ± 4.35 µV) elicited larger N2 amplitudes as compared to high-calorie foods (M = − 2.27 ± 4.02 µV; *t*[57] = 3.69, *p* < 0.001, Cohen’s *d* = 0.48; Fig. 4B). Conversely, high-calorie foods led to a significantly larger P3 (*t*[57] = 5.96, *p* < 0.001, Cohen’s *d* = 0.78; Fig. 4C) and LPP amplitudes (*t*[57] = − 6.33, *p* < 0.001, Cohen’s *d* = 0.83; Fig. 4D) compared to low-calorie foods. Particularly, both P3 and LPP amplitudes were significantly higher in the high-calorie (P3: M = 1.49 ± 4.44 µV; LPP: M = 0.19 ± 4.13 µV) condition than in the low-calorie condition (P3: M = − 0.48 ± 3.66 µV; LPP: M = − 2.33 ± 4.21 µV). The mean differences between high- and low-calorie were consistent for each electrode site (see details in Supplementary Table 2).

HDDM regression analysis was conducted to explore the associations between these ERPs and cognitive parameters (i.e., *v* and *z*). The results revealed non-significant main effects of ERPs on starting point *z* (all *ps* > 0.05; see Supplementary Table 3). However, our research observed that LPP amplitude negatively predicted the drift rate *v* (*B* = − 0.007, 95%HDI [− 0.01, − 0.001]), suggesting the larger LPP amplitude is observed for binary comparisons with the smaller drift rate (Participants may need more evidence to make a decision).

**Fig. 5:**
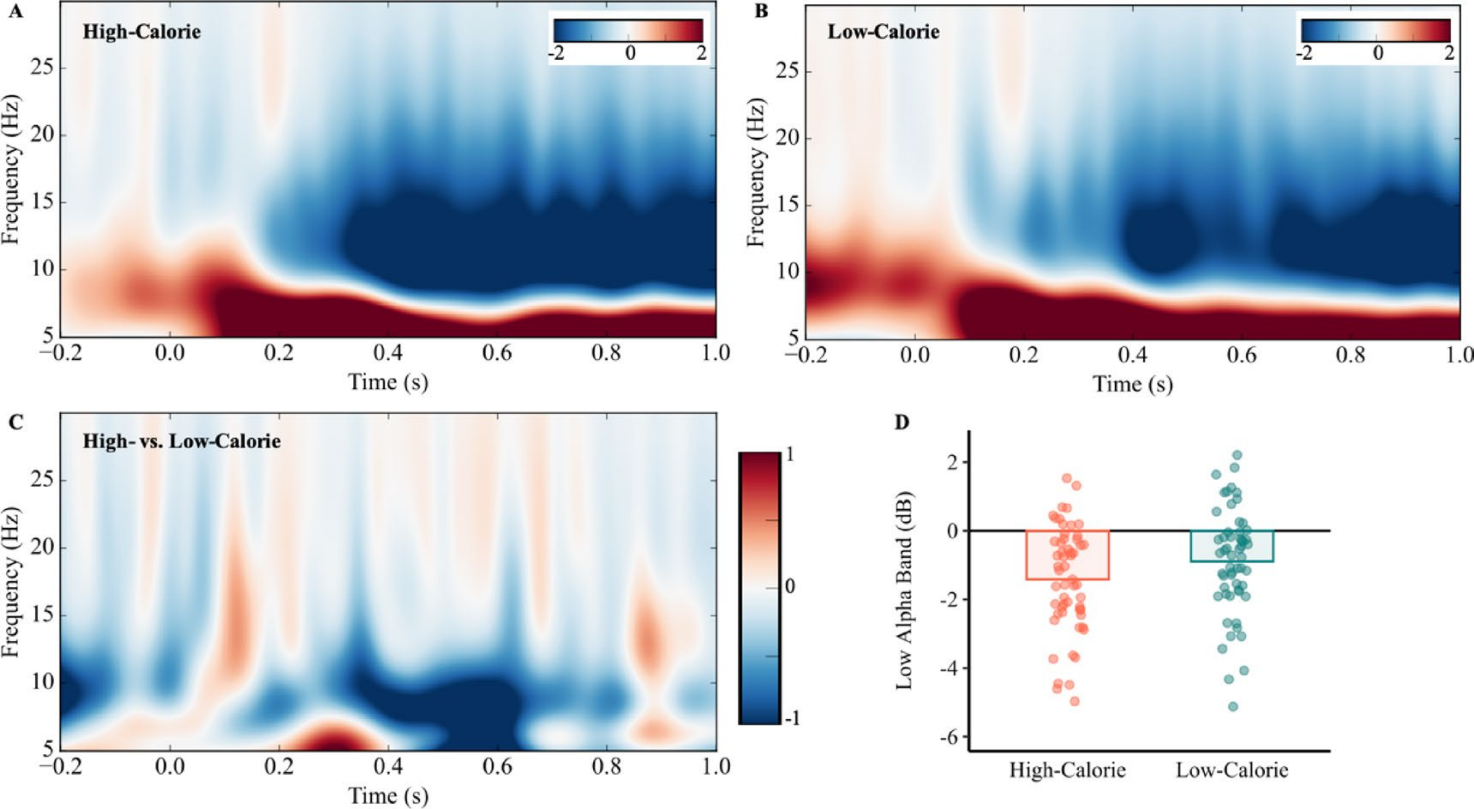
Power differences of frontal alpha event-related desynchronization between the high- and low-calorie conditions. Time-frequency representation of EEG activity for the high-calorie (**A**), low-calorie (**B**), and their difference (**C**) condition. (**D**) Event-related desynchronization (ERD) in the low-alpha band was significantly greater in the high-calorie condition compared to the low-calorie condition.

To investigate the oscillatory neural dynamics underlying participants’ preference for outcome uncertainty in food choices, the cluster-based permutation of neural oscillations within 5–30 Hz was conducted in a 0–1000 ms time window at the fronto-central electrode cluster (F1, Fz, F2, FC1, FCz, FC2). The permutation identified a significant negative cluster covering lower-alpha (8–10 Hz) at 300–600 ms at the fronto-central electrode cluster (Supplementary Fig. 6). Specifically, larger low-alpha ERD was observed during food decision-making with respect to high-calorie foods (M = − 1.19 ± 1.67 dB), compared to low-calorie foods (M = − 0.46 ± 1.58 dB; *t*[57] = 4.10, *p* < 0.001, Cohen’s *d* = 0.54; Fig. 5). The mean differences between high- and low-calorie were consistent for each electrode site (see details in Supplementary Table 4). According to HDDM regression, neither the main effect of lower-alpha ERD on drift rate nor starting point (all *ps* > 0.05) was observed.

## Discussion

The present research reveals that participants tended to choose the uncertain options in the low- (vs. high-) calorie condition, because they were associated with a higher subjective value (i.e., expected to be tastier) as compared to the certain options. This process of value-based decision-making was further demonstrated by the HDDM parameters. Specifically, the drift rate was larger in the low- (vs. high-) calorie condition. Besides, significant differences were observed between the high- and low-calorie conditions in terms of frontal N2, P3, and LPP, and alpha ERD. These findings contribute to our understanding of food-related decision-making by demonstrating that outcome uncertainty can modulate subjective value, particularly in the context of food choices.

### Joint effect of uncertainty-of-outcome and calorie content on food preference

Participants were more inclined to choose the uncertain options than the certain options. Prior research has shown that uncertain options elicit positive expectations^11,12^, anticipation of enjoyment and positive surprise^15,16^, and an inherent curiosity to resolve it^6^. In line with that, the current research provides direct evidence in terms of the relatively larger subjective reward value of uncertain options in decision-making. Specifically, participants’ preference for uncertain options stems from a comparative evaluation of the subjective value of both certain and uncertain choices, highlighting the crucial role of subjective reward value in driving preference for uncertain options.

Moreover, participants’ preference for uncertain options was stronger in the low-calorie condition as compared to the high-calorie condition, suggesting an interaction between the calorie content and uncertainty-of-outcome. Given the binary-choice task, participants may be engaging in avoidance-driven decision-making in the low-calorie condition, where the alternative uncertain option is preferred, to avoid the less appealing low-calorie certain option^19,63^. Such decision-making may reflect a form of risk-seeking, as suggested in prior research^64^. Specifically, people tend to approach appealing foods, such as high-calorie foods, while avoiding both disgusting and neutral foods, such as vegetables^65^. As survival during ancient times was dependent upon a person’s ability to efficiently identify and gather high-quality resources^66^, high- (versus low-) calorie foods are generally considered more appealing thus motivating people to approach them. However, recent studies have challenged this notion, identifying a stronger approach bias towards low-calorie foods as compared to high-calorie options due to the reward-based learning mechanism^20^. Specifically, repeated exposure to the long-term benefits of low-calorie foods can lead to the implicit association of such foods with positive outcomes, such as improved health. This association can trigger automatic behavioural responses, increasing the likelihood of choosing low-calorie options.

However, our HDDM analysis demonstrated a larger drift rate in the low- (vs. high-) calorie condition, thus suggesting a valuation bias favoring the uncertain options particularly within a low-calorie condition, rather than a response bias^67^. According to the literature that has been published to date, drift rate and starting point in the HDDM model are thought to reflect valuation and response bias, respectively^68,69^. Moreover, the relatively larger difference of subjective value (i.e., the difference between expected value of uncertain options and subjective value of certain options) facilitated faster decision-making in the low- (vs. high-) calorie condition. According to the dual process theory^17,18^, uncertainty-of-outcome may activate the impulsive system by enhancing the subjective value of foods, leading to a more rapid decision in the low-calorie condition (less response time in our study). However, when the subjective values of two options are closer, it tends to intensify cognitive conflict and thus increase the difficulty of the decision-making process^41^. As larger drift rates indicate the faster speed of evidence accumulation in decision-making^68,69^, our research provided evidence that larger differences in subjective value are associated with a larger drift rate, thus leading to relatively faster decision-making.

Additionally, after the uncertain option was revealed, it was rated as being less tasty as compared to the certain option, regardless of its calorie content. Specifically, when the actual outcome fails to meet the consumer’s expectations, a negative disconfirmation may result, potentially triggering a negative emotional response^70^. Aligning with this, the results reported here show that the greater the disconfirmation regarding the uncertain option’s tastiness upon its revelation (i.e., the less tasty the revealed uncertain option), the less likely participants were to choose the uncertain option in the next round. However, participants’ overall selection proportions fluctuated around 54.89% for the high-calorie condition and 74.30% for low-calorie condition (see Supplementary Fig. 1). One possible explanation is the inherent motivation driven by curiosity to discover what’s inside the box. Specifically, people experience curiosity when they become aware of an information gap, which in turn motivates them to take action to fill that gap^6^. Curiosity driven by uncertainty-of-outcome acts as a strong motivator, leading people to resolve uncertainty even when anticipating negative consequences—a phenomenon known as the Pandora effect^6,15^. This is especially true when there are no actual consequences for a disconfirmation of expectations, and the loss has less impact on their lives. Particularly, people may be less willing to choose an uncertain option if the outcome holds significant value for them (e.g., booking a hotel for their honeymoon), even when the general quality range of the hotel is known. For daily meals, a less tasty option is more acceptable because the perceived loss is small.

### Neural responses underlying food valuation in the binary-choice task

Frontal cortex plays a crucial role in processing the subjective reward of food^2,23,24^. Using the binary-choice task, the current research detected significant differences of ERPs and EROs in the frontal sites. Specifically, larger frontal N2 amplitudes were observed in the low- (vs. high-) calorie condition. In line with our findings, frontal N2 is demonstrated its role in economic valuation processing^44^, with larger amplitudes is observed in response to options that are perceived as having a lower subjective value^34^. This suggests that N2 is involved in the early stage of reward processing during value-based decision-making. Moreover, larger frontal N2 amplitudes has been observed in inhibiting the high-calorie (vs. low-calorie and neutral items) food during the go/no-go task^37^. Particularly, the N2 amplitudes are even larger in no-go trials for high-calorie foods than in go trials for low-calorie foods^38^. However, in binary-choice tasks, choosing between two aversive options (i.e., selecting one from two loss options) elicits larger N2 amplitudes compared to making decisions between two gain options^45,71^. Given the less palatable and lower hedonic value of low-calorie foods, participants may face a stronger conflict when choosing in a low-calorie binary task compared to high-calorie foods, thus showing a larger frontal N2 amplitude.

Additionally, larger P3 and LPP amplitudes are observed in the high-calorie (vs. low-calorie) condition. In past studies, the posterior parietal P3 and LPP amplitude reflect the allocation of sustained attentional resources and visual processing to stimuli^40,72,73^. Recently, frontal ERPs correlated with subjective stimulus value are observed approximately 300–500 ms after stimuli onset^74^. Thus, the N2, P3, and LPP components reflect distinct neural dynamics across stages of value-based decision-making, including early conflict detection and subsequent cognitive processing. Intriguingly, our research revealed a negative association between frontal LPP amplitude and drift rate, suggesting that increased cognitive resources (reflected in a larger frontal LPP amplitude) were allocated during the value-based decision-making. Specifically, frontal LPP amplitudes have been regarded as providing an objective index of later-stage cognitive effort^75^, particularly associating with reflective processing^76^. Based on the dual process theory, the reflective system is slow and requires more cognitive effort^17^. Thus, the frontal LPP amplitudes may reflect the cognitive effort involved in value comparison processes, particularly when both food options are appealing and more evidence is needed to make a decision. However, since high-calorie trials typically exhibited both larger LPP amplitudes and lower drift rates, the observed cross-condition association may reflect differences between calorie content conditions.

### Bottom-up modulation underlying value-based decision-making

High-calorie (vs. low-calorie) foods elicited larger frontal alpha ERD, suggesting alpha activities are modulated by subjective value (i.e., calorie content). Particularly, frontal alpha ERD reflects bottom-up cognitive processing^55^. Stronger alpha ERD, especially in posterior brain regions, is thought to reflect stronger demands on the visual processing system^77^. However, frontal alpha power is thought to reflect executive top-down control^78^. In the posterior areas, stronger alpha event-related desynchronization is also found to be correlated with greater evidence accumulation^79^. According to the dual process theory^17^, participants may require more evidence and effort in value comparison during the binary-choice task, especially when two high-calorie options have similar subjective values, making decisions difficult. The frontal alpha power reflects enhanced evidence accumulation during dietary decisions, where weaker frontal alpha power is associated with a larger drift rate^53^. Hence, frontal alpha ERD may reflect the cognitive efforts involved in accumulating evidence during value comparison, leading to larger frontal alpha ERD in the high-calorie (vs. low-calorie) condition.

### Implications

Eating healthily is often perceived as boring, which can lead to failure in adhering to regular health activities. Uncertainty-of-outcome may be used to promote healthier choices and enhance sustained engagement in healthy behaviours. Specifically, uncertainty-of-outcome not only directly promotes the subjective value of the activity, but it also serves an effective approach against negative experience, promoting behavioural maintenance^6^. Our research revealed that individuals presented with low-calorie options were significantly more likely to select choices characterized by uncertainty-of-outcome (approximately 75%, see Supplementary Fig. 1). This suggests that incorporating uncertainty-of-outcome into health-promotion products could serve as a strategy for facilitating the maintenance of healthy behaviours.

Additionally, repeatedly eating identical dishes can lead to sensory-specific satiety, causing boredom or fatigue with the food, even if it was initially enjoyable^70,80^. Introducing uncertainty-of-outcome in daily meals, achieved by randomly selecting dishes from a predetermined set of options, could be a strategy for enhancing positive expectations and enjoyment. Moreover, uncertain promotion is actually being used in reducing food waste^81^. Specifically, restaurants have incorporated uncertainty into their promotions by offering mystery boxes containing surplus food or items nearing expiry. These boxes offer customers an element of surprise as the exact contents remain unknown. Notably, according to our findings, incorporating uncertainty can transform the perception of food that might have been initially disregarded, creating a positive consumer experience. Ultimately, this approach presents a compelling opportunity to increase sales while simultaneously contributing to the reduction of food waste.

### Limitations

In this research, participants exhibited a stronger preference for uncertain options, leading to an insufficient number of trials for neural comparisons between certain and uncertain options. By increasing the number of trials for each condition, future studies could investigate differences in neural activity when choosing between uncertain and certain options. It may help us better understand the neural mechanisms underlying the choice of uncertain and certain option. Moreover, as the number of trials increased, the participants’ response times were reduced (see details in Supplementary Fig. 4), suggesting that participants became more efficient at the repeated task (i.e., a learning effect). However, the tastiness evaluation of uncertain options may vary over trials due to the participants’ learning capacity and the feedback from prior decisions. Furthermore, participants rated the tastiness of each food item in advance using a 4-point scale, allowing the researchers to estimate perceived tastiness for each certain option during the binary choice task. However, this evaluation may have influenced participants’ decision-making strategies. According to Bayesian inference, people tend to update their beliefs about latent variables in generative models by incorporating new information^82^. Future studies should analyze the expected value of uncertain options using cognitive modeling, such as Bayesian inference, and further investigate the relationship between learning capacity and preferences regarding uncertainty-of-outcome. Additionally, participants made decisions in either high-calorie versus low-calorie conditions without receiving actual feedback on their choices. Participants’ preferences may be influenced by real food options, particularly in contexts involving reward uncertainty. Future studies should consider employing the Becker-DeGroot-Marschak (BDM) auction task to gain a more comprehensive understanding of how calorie content and uncertainty-of-outcome interact to influence food preferences.

## **Conclusions**

Participants exhibited a stronger preference for uncertain options in the low- (vs. high-) calorie condition, as evidenced by value comparison results showing a larger drift rate in the HDDM. Moreover, our research detected a larger frontal N2 amplitude and smaller frontal P3 and LPP amplitudes in the low- (vs. high-) calorie condition during the binary-choice task. Notably, frontal LPP amplitudes were negatively correlated with drift rate, suggesting that greater cognitive effort was made in response to large evidence accumulation. Additionally, high-calorie foods elicited larger frontal alpha ERD than low-calorie foods, suggesting alpha oscillatory activities are modulated by calorie content.

## Supplementary Information

Below is the link to the electronic supplementary material.

Supplementary Material 1

## Data Availability

The datasets and code used in the current study are available from the corresponding author on reasonable request.
