# Supplementary material for: Joint effect of uncertainty-of-outcome and calorie content on food preference
Source: Sci Rep. 2026 Feb 28;16:11471. doi: 10.1038/s41598-026-41632-x (PMC13056920; doi:10.1038/s41598-026-41632-x)
Supplement: Supplementary file 1 — Supplementary Material 1 [file 41598_2026_41632_MOESM1_ESM.docx]

**Appendix. Supplementary materials**

Supplementary Table. 1 **1**

Supplementary Table. 2 **2**

Supplementary Table. 3 **3**

Supplementary Table. 4 **4**

Supplementary Fig. 1 **5**

Supplementary Fig. 2 **6**

Supplementary Fig. 3 **7**

Supplementary Fig. 4 **8**

Supplementary Fig. 5 **9**

Supplementary Fig. 6 **10**

Supplementary Table. 1

*Model fitness and estimated parameters of HDDMs*

| Model | Model Fitness | |  | Estimated Parameters, 95%HDI | | | | | | | |
| --- | --- | --- | --- | --- | --- | --- | --- | --- | --- | --- | --- |
|  | Gelman-Rubin R | DIC |  | *v*  *High versus Low Calorie* | | *z*  *High versus Low Calorie* | | *a*  *High versus Low Calorie* | | *τ*  *High versus Low Calorie* | |
| 4-Parameter  (*v, z, a, τ*) | 1.0015 | 5780.32 |  | 0.19  [0.02,0.37] | 0.87  [0.70,1.05] | 0.48  [0.47,0.51] | 0.51  [0.49,0.53] | 1.47  [1.40,1.55] | 1.57  [1.50,1.65] | 0.64  [0.59,0.70] | 0.58  [0.53,0.64] |
| 2-Parameter*  (*v, z*) | 1.0006 | 5818.00 |  | 0.19  [0.02,0.36] | 0.83  [0.66,1.00] | 0.48  [0.46,0.50] | 0.51  [0.49,0.53] | 1.53  [1.47, 1.61] | | 0.61  [0.56, 0.66] | |
| 1-Parameter  (*v*) | 1.0003 | 5820.83 |  | 0.16  [-0.003,0.32] | 0.87  [0.71,1.04] | 0.50  [0.48, 0.51] | | 1.54  [1.47, 1.61] | | 0.61  [0.56, 0.66] | |

Note. * indicates the model used in this research;

*v* indicates the drift rate*, z* indicates the starting point*, a* indicates the decision boundary*, τ* indicates the non-decision time.

Supplementary Table. 2

*Amplitude of event-related potentials across electrodes and calorie content*

| Electrodes | Calorie Condition (μV) | |  | Difference Test | | |
| --- | --- | --- | --- | --- | --- | --- |
|  | High | Low |  | t-value | p-value | Cohen’s d |
| N2 (200~300ms) |  |  |  |  |  |  |
| F1 | -2.03 | -2.82 |  | 2.34 | 0.023 | 0.31 |
| FC1 | -1.33 | -2.37 |  | 3.33 | 0.002 | 0.44 |
| Fz | -2.86 | -3.93 |  | 3.33 | 0.002 | 0.44 |
| FCz | -2.27 | -3.52 |  | 3.91 | <0.001 | 0.51 |
| F2 | -2.93 | -4.30 |  | 4.29 | <0.001 | 0.56 |
| FC2 | -2.21 | -3.30 |  | 3.29 | 0.002 | 0.43 |
| P3 (300~400ms) |  |  |  |  |  |  |
| F1 | 1.09 | -0.37 |  | 4.57 | <0.001 | 0.60 |
| FC1 | 2.08 | 0.35 |  | 5.20 | <0.001 | 0.68 |
| Fz | 0.75 | -1.30 |  | 5.67 | <0.001 | 0.74 |
| FCz | 1.62 | -0.56 |  | 5.62 | <0.001 | 0.74 |
| F2 | 1.15 | -1.12 |  | 6.36 | <0.001 | 0.84 |
| FC2 | 2.27 | 0.07 |  | 5.66 | <0.001 | 0.74 |
| LPP (500~700ms) |  |  |  |  |  |  |
| F1 | -0.15 | -2.34 |  | 4.83 | <0.001 | 0.63 |
| FC1 | 0.22 | -2.03 |  | 6.28 | <0.001 | 0.82 |
| Fz | -0.42 | -3.18 |  | 6.32 | <0.001 | 0.83 |
| FCz | 0.17 | -2.43 |  | 5.83 | <0.001 | 0.77 |
| F2 | 0.37 | -2.51 |  | 6.36 | <0.001 | 0.84 |
| FC2 | 0.94 | -1.51 |  | 5.88 | <0.001 | 0.77 |

Supplementary Table. 3

*The effect of neural activities on HDDM parameters*

| Components | Drift Rate *v* | |  | Starting Point *z* | |
| --- | --- | --- | --- | --- | --- |
|  | Beta | 95%HDI |  | Beta | 95%HDI |
| Main Effects of ERPs | |  |  |  |  |
| N2 (200~300ms) | -0.005 | [-0.01, 0.002] |  | 0.0001 | [-0.001, 0.002] |
| P3 (300~400ms) | -0.003 | [-0.01, 0.003] |  | 0.00004 | [-0.001, 0.001] |
| LPP (500~700ms) | -0.007 | [-0.01, -0.001] |  | 0.0001 | [-0.001, 0.001] |
| Interaction Effects of ERPs and Certain Option’s Tastiness | | | | |  |
| N2 (200~300ms) | 0.02 | [0.01, 0.03] |  | 0.001 | [-0.001, 0.002] |
| P3 (300~400ms) | -0.01 | [-0.02, -0.005] |  | 0.0001 | [-0.001, 0.001] |
| LPP (500~700ms) | 0.001 | [-0.005, 0.01] |  | 0.0001 | [-0.001, 0.001] |
| Lower-Alpha Band | | |  |  |  |
| Main Effects | 0.01 | [-0.007, 0.02] |  | 0.001 | [-0.002, 0.004] |
| Interacted with  Certain Option’s  Tastiness | 0.07 | [0.05, 0.08] |  | -0.001 | [-0.003, 0.002] |

Supplementary Table. 4

*The neural oscillations of low-alpha band across electrodes and calorie content*

| Electrodes | Calorie Condition (dB) | |  | Difference Test | | |
| --- | --- | --- | --- | --- | --- | --- |
|  | High | Low |  | t-value | p-value | Cohen’s d |
| Alpha Power (300~600ms) | |  |  |  |  |  |
| F1 | -1.11 | -0.50 |  | -3.33 | 0.002 | 0.44 |
| FC1 | -1.15 | -0.48 |  | -3.58 | <0.001 | 0.47 |
| Fz | -1.21 | -0.51 |  | -3.89 | < 0.001 | 0.51 |
| FCz | -1.26 | -0.48 |  | -4.26 | < 0.001 | 0.56 |
| F2 | -1.12 | -0.37 |  | -4.10 | <0.001 | 0.54 |
| FC2 | -1.26 | -0.46 |  | -4.22 | <0.001 | 0.55 |

Supplementary Fig. 1

*Group-level perception of choosing uncertain option over rounds*

*
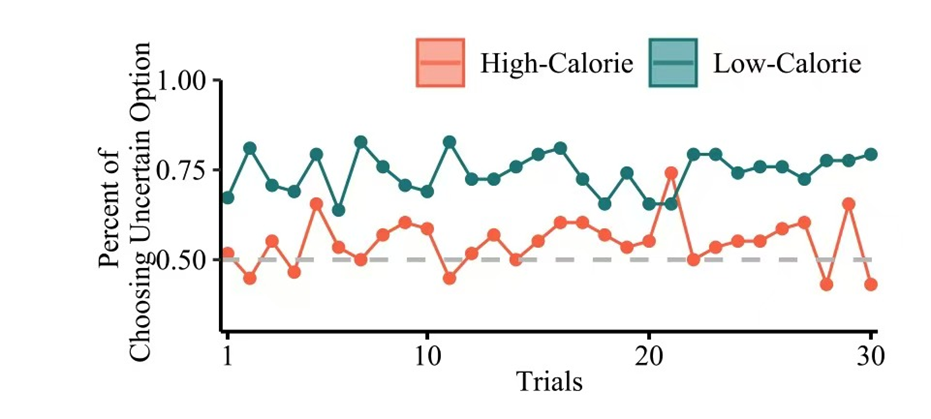
*

Supplementary Fig. 2

*The joint effect of uncertain option’s post-decision tastiness (t round) and certain option’s tastiness (t+1 round) on the food preference (t+1 round)*

*
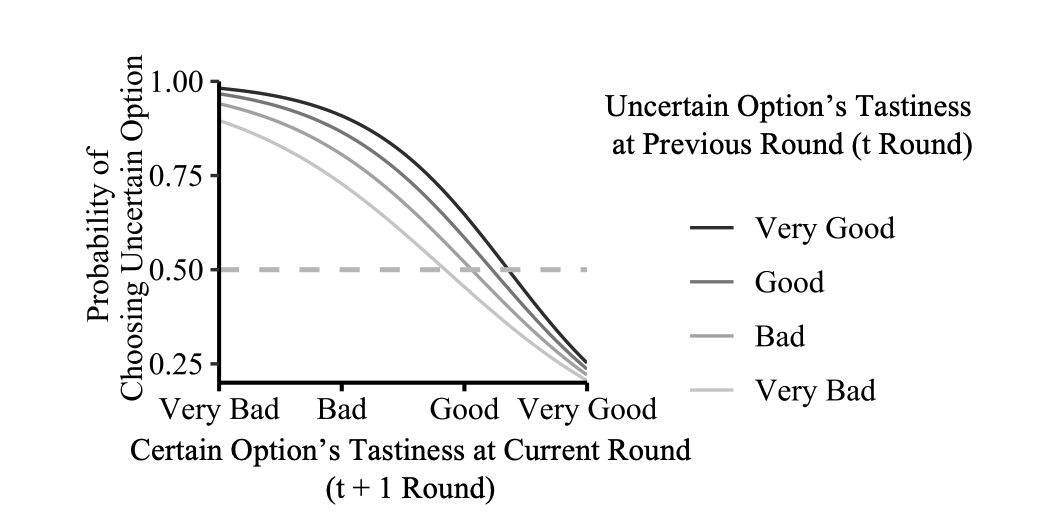
*

*Note.* The post-decision tastiness of uncertain option refers to participants’ ratings of the tastiness of the uncertain option they selected. Our research found that post-decision tastiness of uncertain option interacted with the tastiness of certain option (rated during the food rating task) in predicting participants’ food preferences.

Supplementary Fig. 3

*Group-level mean response time across calorie conditions*


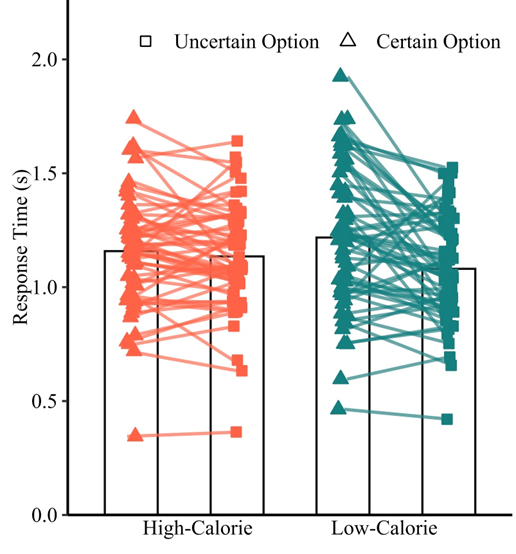


Note. The point indicated the average response time for each individual within the given condition.

Supplementary Fig. 4

*Group-level mean response time over rounds*


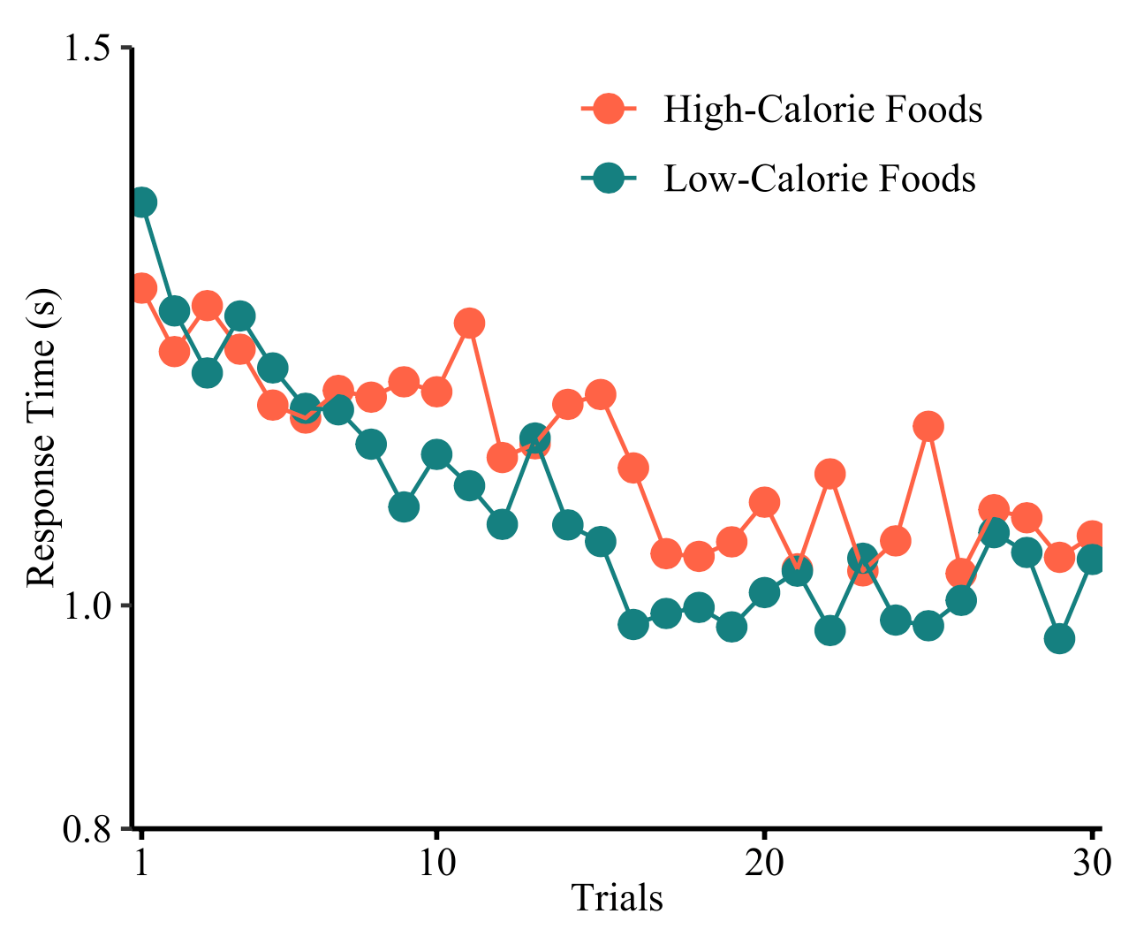


Note. The point indicated the average response time for each condition.

Supplementary Fig. 5

*Posterior prediction check of HDDMs*

Note. In the 4-parameter model, four parameters, including drift rate*,* starting point*,* decision boundary, and the non-decision time, were estimated. In the 2-parameter model, two parameters (drift rate and starting point) were estimated. In the 1-parameter model, drift rate was estimated.

Supplementary Fig. 6

*The results of cluster-based permutation t-test*

*
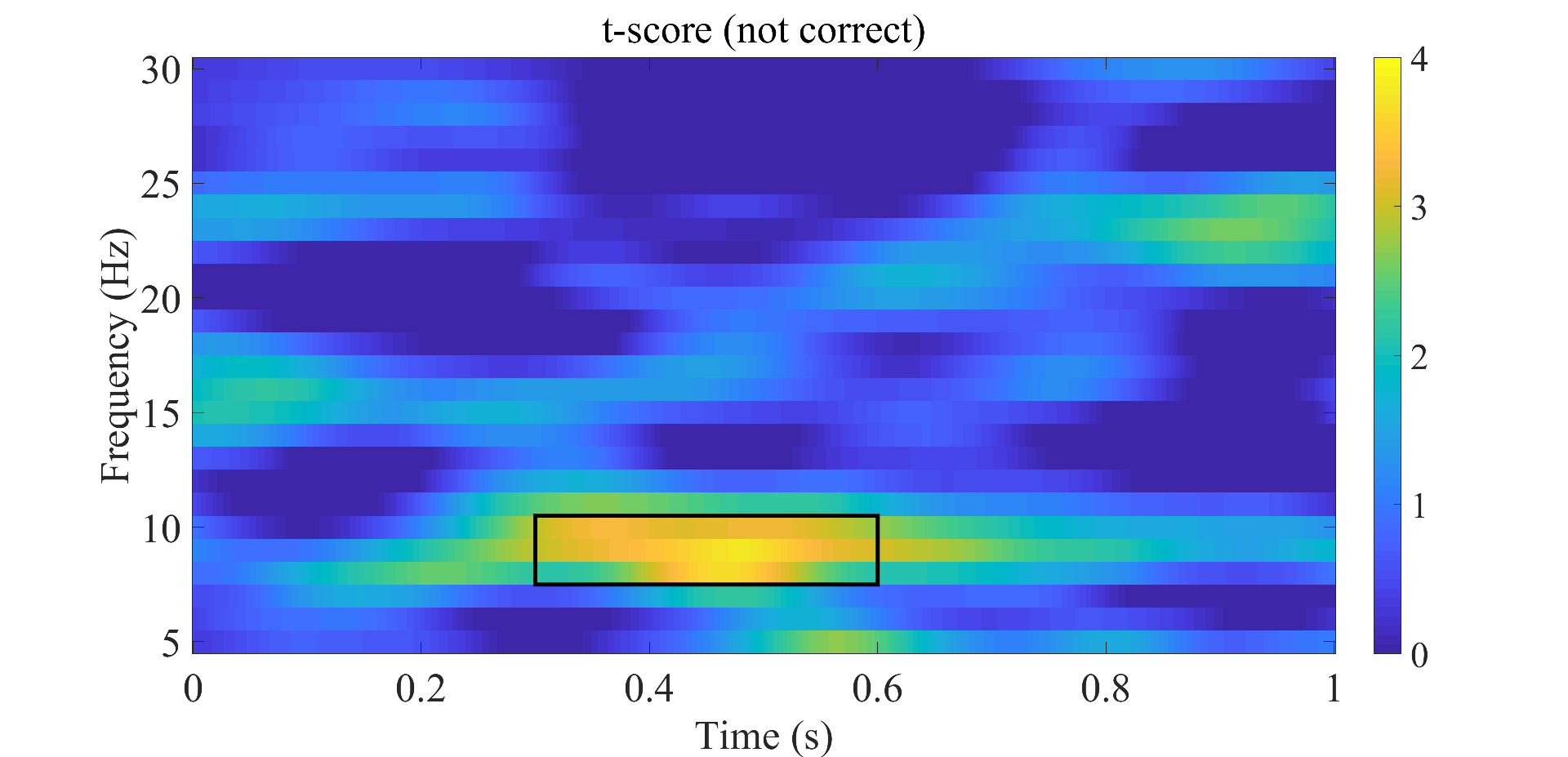
*
